# A novel combined nomogram for predicting severe acute lower respiratory tract infection in children hospitalized for RSV infection during the post-COVID-19 period

**DOI:** 10.3389/fimmu.2024.1437834

**Published:** 2024-07-24

**Authors:** Hai-Feng Liu, Xue-Zu Zhang, Cong-Yun Liu, Wang Li, Wen-Hong Li, Ya-Yu Wang, He-Yun Li, Mei Xiang, Rui Lu, Ting-Yun Yuan, Hong-Min Fu

**Affiliations:** ^1^ Department of Pulmonary and Critical Care Medicine, Yunnan Key Laboratory of Children’s Major Disease Research, Yunnan Medical Center for Pediatric Diseases, Kunming Children’s Hospital, Kunming Medical University, Kunming, Yunnan, China; ^2^ Department of Pediatrics, The People’s Hospital of Lincang, Lincang, Yunnan, China; ^3^ Department of Pediatrics, The People’s Hospital of Baoshan, Baoshan, Yunnan, China; ^4^ Department of Pediatrics, The People’s Hospital of Guandu District, The Fifth People’s Hospital of Kunming, Kunming, Yunnan, China; ^5^ Department of Pediatrics, The People’s Hospital of Lufeng, Lufeng, Yunnan, China; ^6^ Department of Pediatrics, The People’s Hospital of Dali, The Third Affiliated Hospital of Dali University, Dali, Yunnan, China; ^7^ Department of Pediatrics, The First People’s Hospital of Zhaotong, Zhaotong Hospital Affiliated to Kunming Medical University, Zhaotong, Yunnan, China; ^8^ Department of Pediatrics, The People’s Hospital of Honghe, Honghe, Yunnan, China; ^9^ Department of Pediatrics, The People’s Hospital of Wenshan Zhuang & Miao Autonomous Prefecture, Wenshan, Yunnan, China

**Keywords:** RSV, severe acute lower respiratory tract infection, children, nomogram, post-COVID-19 period, machine learning

## Abstract

**Introduction:**

Off-season upsurge of respiratory syncytial virus (RSV) infection with changed characteristics and heightened clinical severity during the post-COVID-19 era are raising serious concerns. This study aimed to develop and validate a nomogram for predicting the risk of severe acute lower respiratory tract infection (SALRTI) in children hospitalized for RSV infection during the post-COVID-19 era using machine learning techniques.

**Methods:**

A multicenter retrospective study was performed in nine tertiary hospitals in Yunnan, China, enrolling children hospitalized for RSV infection at seven of the nine participating hospitals during January–December 2023 into the development dataset. Thirty-nine variables covering demographic, clinical, and laboratory characteristics were collected. Primary screening and dimension reduction of data were performed using Least Absolute Shrinkage and Selection Operator (LASSO) regression, followed by identification of independent risk factors for RSV-associated SALRTI using Logistic regression, thus finally establishing a predictive nomogram model. Performance of the nomogram was internally evaluated by receiver operating characteristic (ROC) curve, calibration curve, and decision curve analysis (DCA) based on the development dataset. External validation of our model was conducted using same methods based on two independent RSV cohorts comprising pediatric RSV inpatients from another two participating hospitals between January–March 2024.

**Results:**

The development dataset included 1102 patients, 239 (21.7%) of whom developed SALRTI; while the external validation dataset included 249 patients (142 in Lincang subset and 107 in Dali subset), 58 (23.3%) of whom were diagnosed as SALRTI. Nine variables, including age, preterm birth, underlying condition, seizures, neutrophil-lymphocyte ratio (NLR), interleukin-6 (IL-6), lactate dehydrogenase (LDH), D-dimer, and co-infection, were eventually confirmed as the independent risk factors of RSV-associated SALRTI. A predictive nomogram was established via integrating these nine predictors. In both internal and external validations, ROC curves indicated that the nomogram had satisfactory discrimination ability, calibration curves demonstrated good agreement between the nomogram-predicted and observed probabilities of outcome, and DCA showed that the nomogram possessed favorable clinical application potential.

**Conclusion:**

A novel nomogram combining several common clinical and inflammatory indicators was successfully developed to predict RSV-associated SALRTI. Good performance and clinical effectiveness of this model were confirmed by internal and external validations.

## Graphical Abstract

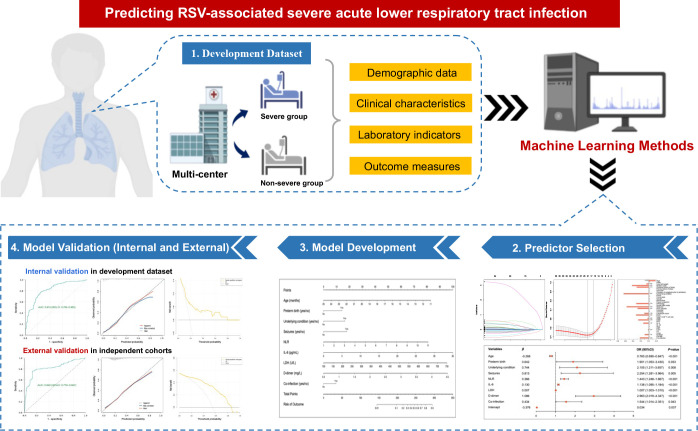

## 1 Introduction

Respiratory syncytial virus (RSV) is well-known as the principal pathogen of acute lower respiratory tract infection (ALRTI) among children (1). Due to its high contagiousness, RSV affects about 70% of infants before the first year of life and almost all children by the age of two years (2). The authoritative epidemiology data indicated that there were estimated 33.0 million [uncertainty range (UR): 25.4–44.6 million] episodes of RSV-attributable ALRTI in 2019 globally, leading to 3.6 million (UR: 2.9–4.6 million) hospitalizations and 101,400 (UR: 84,500–125,200) in-hospital deaths among children aged < 5 years (3). Importantly, about 20.8%–25.5% of RSV-ALRTI will progress to severe ALRTI (SALRTI), which is widely accepted as the major cause of RSV-associated in‐hospital deaths in pediatric population and poses a huge threat to children’s health (4, 5).

The influence of COVID-19 pandemic and its associated containment measures on the seasonal circulation of other respiratory pathogens is a serious concern for public health, even extending beyond the immediate consequences of the SARS-CoV-2 infection (6). A series of respiratory viruses have shown significant resurgences during the post-pandemic period, particularly those with a lack of licensed vaccines for children, including RSV, metapneumovirus, and rhinovirus, etc (6–8). Among these, RSV, the most common cause of pediatric ALRTI, is perhaps the most affected one. Despite the presence of several monoclonal antibodies for pediatric RSV infection, such as Palivizumab (FDA-approved drugs but not currently available in mainland China) and Nirsevimab (the first monoclonal antibody approved for the prevention of RSV in infants in mainland China recently), they have not yet been widely used in the population (9, 10). Based on this and the absence of licensed vaccines as well as the gradual decrease of maternally-derived neutralizing antibodies with increasing age (11), seasonal exposure to RSV is still the predominant way for children to produce immunological protection against RSV infection. Therefore, the susceptibility to RSV in children would theoretically increase due to the lack of seasonal viral exposures during the COVID-19 pandemic, thereby causing more intense rebound after easing the containment measures. In fact, as predicted, upsurges of RSV activity have been reported successively in numerous countries and regions worldwide during the post-COVID-19 era (12–15). Even more worrisome, compared to the pre-COVID-19 period, RSV infection among children during the post-pandemic era showed an enhanced clinical severity, reflected by the increased RSV hospitalization rate and proportion of SALRTI with more pediatric intensive care unit (PICU) admission and longer PICU length of stay (LOS) (8, 12), which may be partially attributed to the waning population immunity for RSV and long-lasting impairment of the immune system in individuals after SARS-CoV-2 infection (16, 17). These potential immunological changes and increased severity in RSV infection during the post-COVID-19 era present a significant challenge for the clinical management of such patients, especially for severe cases.

Considering these grim situations during the post-COVID-19 era, it is urgently needed and of great significance to establish a novel, simple, and accurate prediction tool for SALRTI in children hospitalized for RSV infection. Consequently, this multicenter retrospective study summarized and analyzed the clinical and laboratory characteristics of pediatric RSV inpatients from nine tertiary hospitals in Yunnan, China, during post-COVID-19 period, aiming to develop and validate a nomogram model for predicting RSV-associated SALRTI.

## 2 Methods

### 2.1 Study population and design

**Figure 1:**
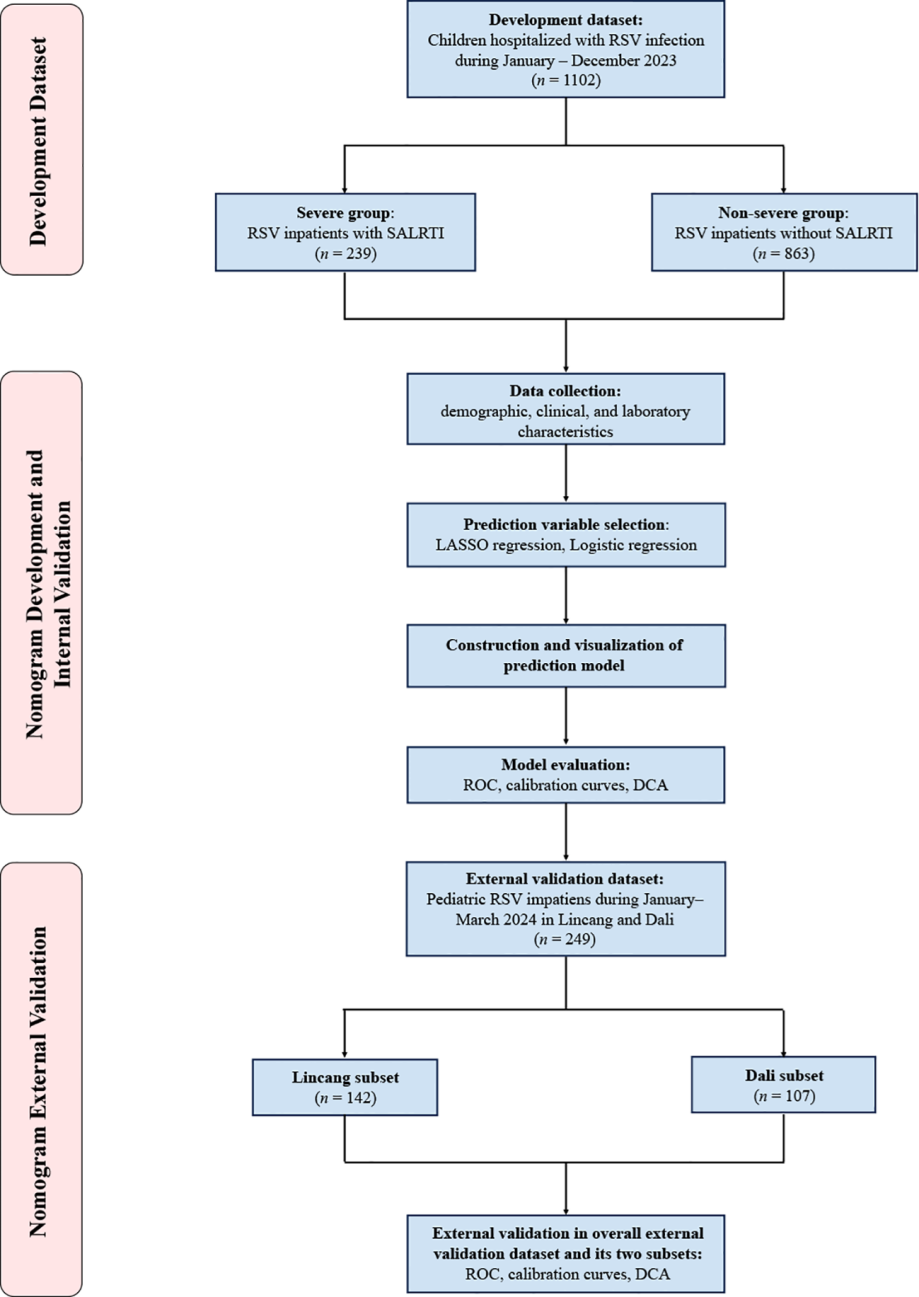
Flow chart of the study.

This was a multicenter study carried out in nine public tertiary hospitals in Yunnan, China. Children (≤ 14 years) hospitalized for RSV infection at seven of the nine participating hospitals in Yunnan between January–December 2023 were retrospectively included into the development dataset of this study and no exclusion criteria were set for this study. All enrolled patients were divided into severe and non-severe groups, depending on the presence or absence of SALRTI, respectively (**Figure 1**).

SALRTI was defined as pneumonia or bronchiolitis accompanied by at least one of the following presentations (18, 19): 1) a reduction in feeding amount to less than half of the normal, dehydration, or refusal to feed; 2) impaired consciousness; 3) manifestations of hypoxemia, including cyanosis, age‐specific tachypnea (≥60 breaths/min for children under 2 months; ≥50 breaths/min for children aged 2 months to 1 year; ≥40 breaths/min for children aged >1 to 5 years; ≥30 breaths/min for children older than 5 years), three concave signs, nasal flaring, grunting, intermittent apnea, or oxygen saturation <88%; 4) persistent high fever for more than 5 days; 5) pulmonary imaging suggesting ≥2/3 unilateral lung infiltration, multilobar pulmonary infiltration, pleural effusion, pneumothorax, atelectasis, pulmonary necrosis, or pulmonary abscess; 6) extrapulmonary complications.

Ethical approval for this study was granted by the Ethics Committees of Kunming Children’s Hospital (approval number: 2023-05-012-K01), who also waived the written informed consent due to the retrospective design of the study.

### 2.2 Potential prediction variables

A total of 39 relevant indicators collected from electronic medical records were used as potential prediction variables, including general characteristics [age, gender, low birthweight, preterm birth, personal and family histories of atopy, non-exclusively breastfeeding (non-EBF)], clinical characteristics (duration of symptoms prior to admission, underlying condition, fever, fever peak, cough, rhinorrhea, nasal congestion, wheezing, acute otitis media, seizures, poor appetite, diarrhea, vomiting), laboratory parameters [leucocyte count, neutrophil-to-lymphocyte ratio (NLR), CD4^+^/CD8*^+^* T cell ratio, erythrocyte sedimentation rate (ESR), c-reactive protein (CRP), procalcitonin (PCT), interleukin 6 (IL-6), IL-10, alanine transaminase (ALT), aspartate transaminase (AST), lactate dehydrogenase (LDH), creatinine (Cr), urea, D-dimer, creatine kinase myocardial band (CK-MB), co-infection], and outcome measures (mechanical ventilation, PICU admission, LOS).

In the above variables, low birthweight was defined as birthweight < 2500 g; preterm birth was defined as birth at < 37 weeks’ gestation; underlying condition included congenital heart disorders, bronchopulmonary dysplasia, pectus excavatum, malignant tumors, inborn errors of immunity, or severe malnutrition; according to the World Health Organization (WHO) (20), EBF was defined as individuals who were fed exclusively with breastmilk in the first 6 months of life, while others were classified as non-EBF. In addition, nasal and throat swabs were collected from patients within 24h of admission for pathogen detection, which was conducted using multiple real-time polymerase chain reaction (RT-PCR) (Adicon, Hangzhou, China) covering RSV, influenza A/H1N1, influenza A/H3N2, influenza B, parainfluenza virus (types 1–3), adenovirus, bocavirus, metapneumovirus, rhinovirus, seasonal coronavirus, *Mycoplasma pneumoniae*, and *Chlamydia pneumoniae*. Co-infection was defined as confirmed RSV infection concurrent to the presence of one or more of the above pathogens. All data were reviewed and cross-checked by two trained Ph.D. students.

### 2.3 Statistical analysis

R software version 3.5.1 (R Foundation for Statistical Computing, Vienna, Austria; https://cran.r-project.org/) was employed for statistical analysis and visualization. A two-tailed *P*-value < 0.05 was considered statistically significant.

#### 2.3.1 Descriptive statistics

The distribution of continuous variables was evaluated using the Shapiro-Wilk test. Continuous variables with skewed distribution were presented as medians [interquartile range (IQR)] and compared using Mann-Whitney U test, while categorical variables were described as frequencies (n) [percentages (%)] and compared using Pearson’s chi-square or Fisher’s exact test. It should be pointed out that median imputation (for continuous variables) and mode imputation (for categorical variables) were adopted to handle missing data.

#### 2.3.2 Candidate predictors selection

To minimize potential multicollinearity and overfitting, Least Absolute Shrinkage and Selection Operator (LASSO) regression, a penalized regression model with L1 regularization, was employed to select candidate prediction variables via the R package ‘*glmnet*’ (https://cran.r-project.org/web/packages/glmnet/). Specifically, LASSO regression shrinks coefficients of irrelevant variables toward zero, while variables with non-zero coefficients will be retained as candidate predictors, thus reducing the data dimensionality. Except for the three outcome measures (mechanical ventilation, PICU admission, LOS), the remaining 36 variables were entered into the LASSO model. The optimal shrinkage parameter lambda was determined through 10-fold cross-validation adopting the built-in function ‘*cv.glmnet*’ from ‘*glmnet*’. In our study, the lambda.1se (within one standard error of minimum lambda) was selected as optimal lambda, since its ability to choose the most concise variables with good predictive performance.

#### 2.3.3 Model development and internal validation

The candidate predictors obtained from LASSO regression were included into multivariable logistic regression analysis, thus determining the independent risk factors for RSV-associated SALRTI and their corresponding regression coefficients (*β*) as well as intercept value. A nomogram was generated based on the results of multivariable logistic regression analysis using the R package ‘*rms*’ (http://cran.r-project.org/package=rms). Then, the patients in the development dataset were randomly divided into 70% as a training set and 30% as an internal validation set utilizing the ‘*caret*’ package (https://cran.r-project.org/web/packages/caret/). Receiver operating characteristic curve (ROC), calibration curve, and decision curve analysis (DCA) were realized using the ‘*pROC*’ (https://cran.r-project.org/web/packages/pROC/), ‘*rms*’ (http://cran.r-project.org/package=rms), and ‘*rmda*’ (https://cran.r-project.org/web/packages/rmda/), respectively, to evaluate performance of the nomogram in both training and internal validation sets.

#### 2.3.4 External validation

For externally validating our nomogram, an external validation dataset was constructed, including two independent pediatric RSV cohorts during January–March 2024. One is the Lincang subset, consisting of 142 inpatients, provided by the People’s Hospital of Lincang, and the other is the Dali subset, consisting of 107 inpatients, provided by the Third Affiliated Hospital of Dali University. The same assessment approaches as used in internal validation, including ROC curve, calibration curve, and DCA, were employed for the external validation of the nomogram.

## 3 Results

### 3.1 General characteristics of patients

A total of 1102 children hospitalized for RSV infection with a median (IQR) age of 15.1 (10.5, 21.6) months and a male proportion of 57.0% (628/1102) were enrolled in the development dataset, comprising 239 (21.7%) in the severe group and 863 (78.3%) in the non-severe group (**Figure 1**). As shown in **Table 1**, the median (IQR) age in the severe group was 11.2 (9.3, 17.9) months, younger than the 17.3 (14.1, 22.5) months in the non-severe group. Meanwhile, compared to the non-severe group, the severe group showed higher proportions of low birthweight (20.1% *vs.* 12.4%, *P* = 0.002), preterm birth (16.7% *vs.* 11.1%, *P* = 0.02), and personal history of atopy (28.9% *vs.* 20.9%, *P* = 0.009).

**Table 1:** Characteristics of patients in development dataset.

Characteristics	Total (n=1102)	Severe (n=239)	Non-severe (n=863)	*P*-value
General characteristics				
Age, months, median (IQR)	15.1 (10.5, 21.6)	11.2 (9.3, 17.9)	17.3 (14.1, 22.5)	<0.001
Male, n (%)	628 (57.0)	139 (58.2)	489 (56.7)	0.679
Low birthweight, n (%)	155 (14.1)	48 (20.1)	107 (12.4)	0.002
Preterm birth, n (%)	136 (12.3)	40 (16.7)	96 (11.1)	0.020
Personal history of atopy, n (%)	249 (22.6)	69 (28.9)	180 (20.9)	0.009
Family history of atopy, n (%)	168 (15.2)	45 (18.8)	123 (14.3)	0.082
Non-EBF, n (%)	505 (45.8)	116 (48.5)	389 (45.1)	0.342
Clinical characteristics				
Duration of symptoms prior to admission, days, median (IQR)	1.0 (1.0, 1.0)	1.0 (1.0, 2.0)	1.0 (1.0, 1.0)	0.581
Underlying condition, n (%)	163 (14.8)	51 (21.3)	112 (13.0)	0.001
Fever, n (%)	582 (52.8)	136 (56.9)	446 (51.7)	0.152
Fever peak, °C, median (IQR)	37.9 (37.2, 38.5)	38.1 (37.7, 39.2)	37.8 (37.1, 38.3)	0.017
Cough, n (%)	993 (90.1)	219 (91.6)	774 (89.7)	0.373
Rhinorrhea, n (%)	495 (44.9)	115 (48.1)	380 (44.0)	0.261
Nasal congestion, n (%)	452 (41.0)	103 (43.1)	349 (40.4)	0.460
Wheezing, n (%)	448 (40.7)	111 (46.4)	337 (39.0)	0.039
Acute otitis media, n (%)	284 (25.8)	66 (27.4)	218 (25.3)	0.461
Seizures, n (%)	131 (11.9)	41 (17.2)	90 (10.4)	0.004
Poor appetite, n (%)	351 (31.9)	79 (33.1)	272 (31.5)	0.652
Diarrhea, n (%)	56 (5.1)	11 (4.6)	45 (5.2)	0.703
Vomiting, n (%)	72 (6.5)	17 (7.1)	55 (6.4)	0.682
Laboratory findings, median (IQR)				
Leukocyte count, ×10^9^/L	13.8 (11.0, 16.7)	16.4 (13.2, 19.8)	12.9 (10.7, 15.6)	<0.001
NLR	4.2 (3.5, 6.2)	5.6 (3.9, 8.8)	4.0 (3.3, 5.9)	<0.001
CD4^+^ /CD8^+^ T cell ratio	1.2 (0.8, 1.5)	1.1 (0.7, 1.3)	1.2 (1.0, 1.6)	0.063
ESR, mm/H	21.0 (14.0, 29.0)	23.0 (16.0, 33.0)	20.0 (13.0, 27.0)	0.182
CRP, mg/L	12.3 (8.9, 19.6)	16.3 (14.8, 22.5)	11.4 (8.1, 14.3)	<0.001
PCT, ng/mL	0.4 (0.1, 1.2)	0.5 (0.3, 1.6)	0.4 (0.2, 0.9)	0.032
IL-6, pg/mL	19.5 (12.4, 32.8)	22.8 (18.1, 36.7)	17.9 (11.2, 30.5)	0.002
IL-10, pg/mL	16.8 (13.7, 21.3)	21.4 (17.6, 27.0)	15.6 (13.3, 20.1)	<0.001
ALT, U/L	24.0 (19.0, 33.0)	24.0 (20.0, 41.0)	24.0 (19.0, 32.0)	0.416
AST, U/L	26.0 (19.0, 35.0)	28.0 (20.0, 38.0)	26.0 (19.0, 34.0)	0.337
LDH, U/L	398.6 (351.2, 568.9)	402.7 (369.1, 577.2)	395.8 (346.3, 552.1)	0.146
Cr, umol/L	31.6 (26.3, 38.2)	34.5 (29.3, 41.2)	30.4 (25.7, 36.9)	0.484
Urea, mmol/L	3.0 (2.1, 4.5)	3.1 (2.6, 4.8)	3.0 (2.0, 4.4)	0.391
D-dimer, mg/L	1.0 (0.8, 1.3)	1.3 (1.0, 1.6)	1.0 (0.8, 1.2)	<0.001
CK-MB, U/L	51.0 (41.4, 60.8)	54.7 (44.1, 69.6)	50.2 (40.5, 58.3)	0.307
Co-infection, n (%)	439 (39.8)	110 (46.0)	329 (38.1)	0.027
Outcome measures				
Mechanical ventilation, n (%)	86 (7.8)	86 (36.0)	0	<0.001
PICU admission, n (%)	101 (9.2)	101 (42.3)	0	<0.001
LOS, days, median (IQR)	6.0 (4.0, 8.0)	7.0 (6.0, 9.0)	6.0 (4.0, 7.0)	<0.001

ALT, alanine aminotransferase; AST, aspartate aminotransferase; CK-MB, creatine kinase myocardial band; Cr, creatinine; CRP, C-reactive protein; ESR, erythrocyte sedimentation rate; IL-6, interleukin 6; IQR, interquartile ranges; LDH, lactate dehydrogenase; LOS, length of hospital stay; NLR, neutrophil-to-lymphocyte ratio; Non-EBF, non-exclusively breastfeeding; PCT, procalcitonin; PICU, pediatric intensive care unit.

### 3.2 Clinical and laboratory characteristics

For the entire development dataset (**Table 1**), cough (90.1%), fever (52.8%), rhinorrhea (44.9%), nasal congestion (41.0%), and wheezing (40.7%) were the most common clinical manifestations, accompanied by a series of abnormal laboratory indicators (**Table 1**). Noteworthily, there were significant differences regarding the clinical and laboratory characteristics between the severe and non-severe groups. Compared to the non-severe group, more frequent underlying condition, wheezing, and seizures, as well as higher fever peak were observed in the severe group, with higher levels of leukocyte count, NLR, CRP, PCT, IL-6, IL-10, D-dimer, and more frequent co-infection (all *P* < 0.05). In terms of outcome measures, 86 (36.0%) and 101 (42.3%) patients in the severe group required mechanical ventilation and PICU admission, respectively, while no patients in the non-severe group needed theses. Meanwhile, the severe group showed a longer LOS than that in the non-severe group [7.0 (6.0, 9.0) *vs.* 6.0 (4.0, 7.0) days; *P* < 0.001].

### 3.3 Variable selection and development of the nomogram

**Figure 2:**
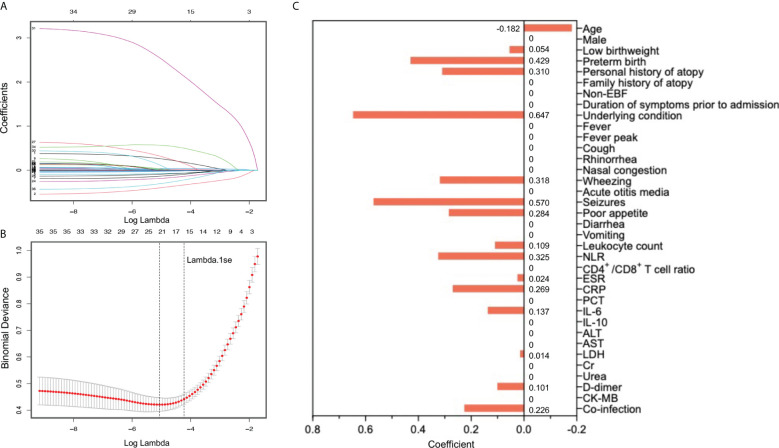
Candidate predictor selection using LASSO regression. **(A)** LASSO coefficient profiles of all 39 potential prediction variables. **(B)** and **(C)** Tuning parameter (lambda) selection in LASSO model using 10-fold cross-validation based on one standard error of the minimum criteria (lambda.1se). Sixteen variables with non-zero coefficients were selected as candidate predictors. LASSO, Least Absolute Shrinkage and Selection Operator.

**Figure 3:**
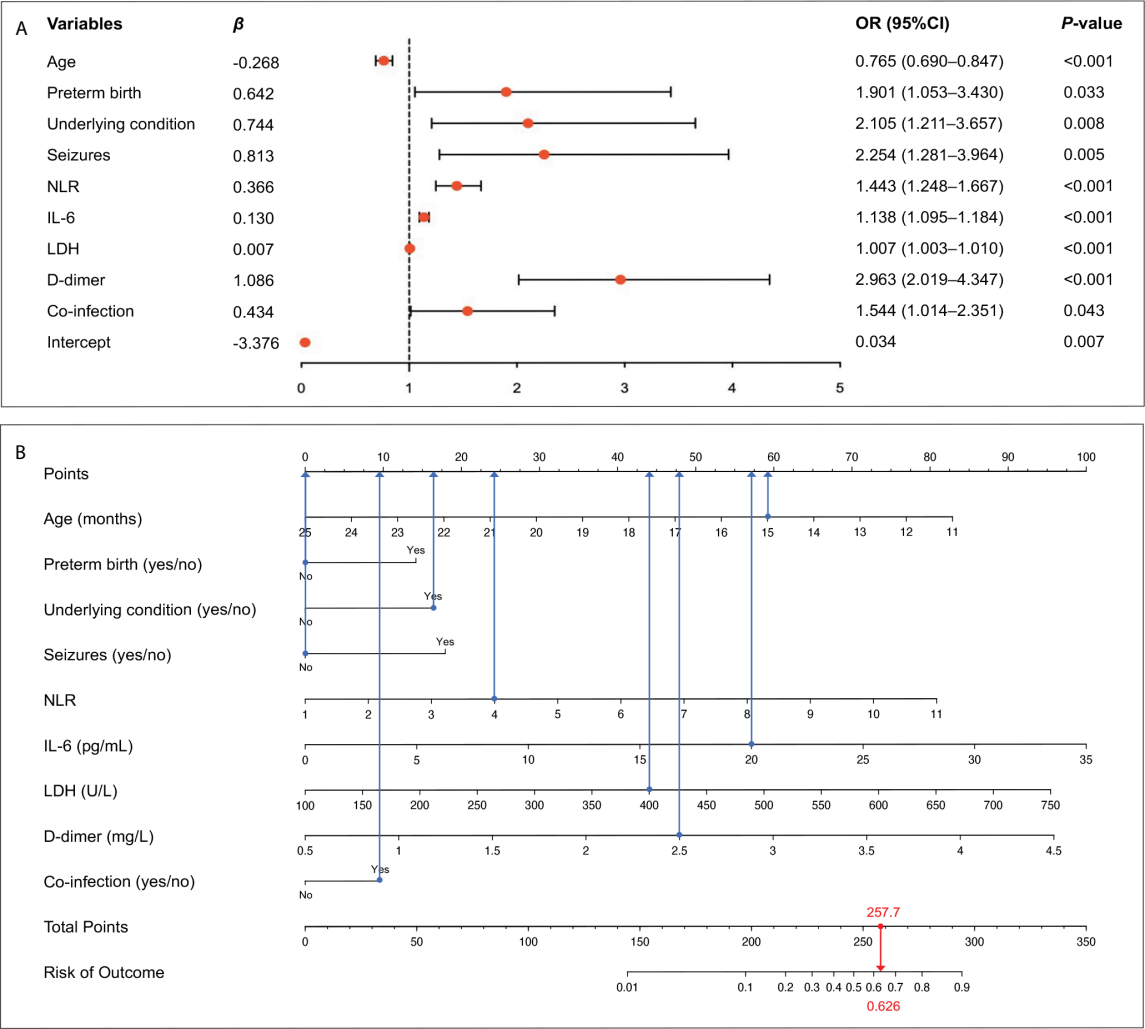
Identification of independent risk factors and construction of prediction nomogram. **(A)** Nine variables, including age, preterm birth, underlying condition, seizures, NLR, IL-6, LDH, D-dimer, and co-infection, were confirmed to be independently associated with RSV-SALRTI. **(B)** A prediction nomogram combining these nine independent predictors was established. IL-6, interleukin 6; LDH, lactate dehydrogenase; NLR, neutrophil-to-lymphocyte ratio; RSV, respiratory syncytial virus; SALRTI, severe acute lower respiratory tract infection.

Since the aim of this study was to develop a prediction model for severe RSV infection, three outcome measures (mechanical ventilation, PICU admission, LOS) representing the disease severity were excluded and the remaining 36 variables were included into the LASSO regression. Based on the value of lambda.1se, 16 variables with non-zero coefficients were selected as candidate predictors, including age, low birthweight, preterm birth, personal history of atopy, underlying condition, wheezing, seizures, poor appetite, leucocyte count, NLR, ESR, CRP, IL-6, LDH, D-dimer, and co-infection (**Figures 2A–C**). These 16 candidate predictors were entered into multivariable logistic regression, eventually identifying nine independent risk factors, including age, preterm birth, underlying condition, seizures, NLR, IL-6, LDH, D-dimer, and co-infection (**Figure 3A**). The logistic regression equation for predicting RSV-associated SALRTI was as follows: Logit (*P*) = −3.376 − 0.268 × Age (month) + 0.642 × Preterm birth (yes) + 0.744 × Underlying condition (yes) + 0.813 × Seizures (yes) + 0.366 × NLR + 0.130 × IL-6 (pg/mL) + 0.007 × LDH (U/L) + 1.086 × D-dimer (mg/L) + 0.434 × Co-infection (yes). To make the prediction model more intuitive and convenience, this model was visualized as a nomogram integrating these nine independent predictors (**Figure 3B**). For example, in our nomogram, a 15-month-old (59.1 points) RSV inpatient without preterm birth history (0 point) and seizures (0 point) had underlying condition (16.3 points), a NLR level of 4.0 (23.8 points), an IL-6 level of 20.0 pg/mL (57.4 points), a LDH level of 400.0 U/L (43.9 points), a D-dimer level of 2.5 mg/L (47.7 points), and co-infection (9.5 points). A total point of 257.7 would be calculated in this patient and the corresponding predicted risk of SALRTI is 0.626.

### 3.4 Internal validation of the nomogram

**Figure 4:**
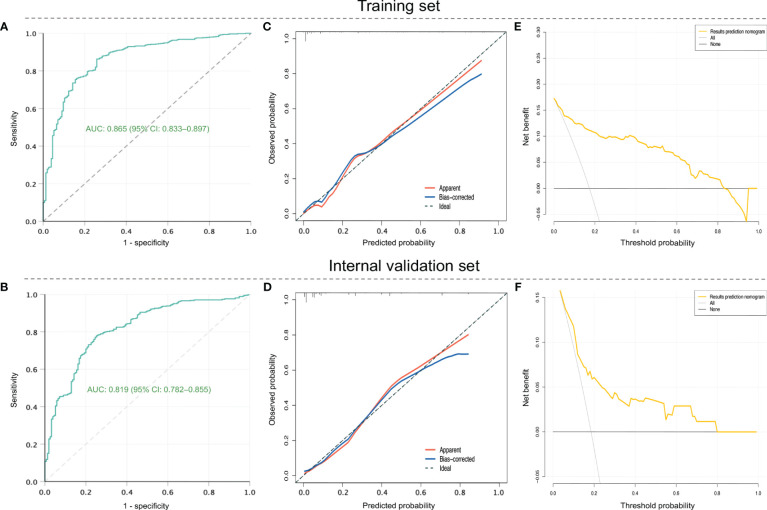
Assessment of the prediction nomogram in training and internal validation sets in the development dataset. **(A)** and **(B)** ROC curves. **(C)** and **(D)** Calibration curves. **(E)** and **(F)** DCA. ROC, receiver operating characteristic; DCA, decision curve analysis.

For internal validation, patients in the development dataset were divided into training and internal validation sets according to the ratio of 7:3. The ROC curves of the nomogram indicated an AUC of 0.865 (95%*CI*: 0.833–0.897, *P* < 0.001), with a sensitivity of 75.8% as well as a specificity of 80.8% in the training set (**Figure 4A**), and showed an AUC of 0.819 (95%*CI*: 0.782–0.855, *P* < 0.001), with a sensitivity of 78.1% as well as a specificity of 76.2% in the internal validation set (**Figure 4B**), suggesting a good discrimination. As shown in **Figures 4C, D**, the nomogram had favorable calibration according to the Hosmer–Lemeshow test that was graphically represented by calibration curves, which demonstrated good consistency between the nomogram-predicted and actual probabilities of RSV-associated SALRTI in both training and internal validation sets (both *P* > 0.05). DCA, taking the threshold probability as the abscissa and the net benefit rate as the ordinate, was also conducted to assess the clinical usefulness of this nomogram. According to **Figures 4E, F**, the nomogram could provide greater net benefits than the “all” and “none” schemes in both training (threshold probability: 0.00–0.83) set and internal validation set (threshold probability: 0.00–0.80), revealing a great potential for clinical utility.

### 3.5 External validation of the nomogram

**Figure 5:**
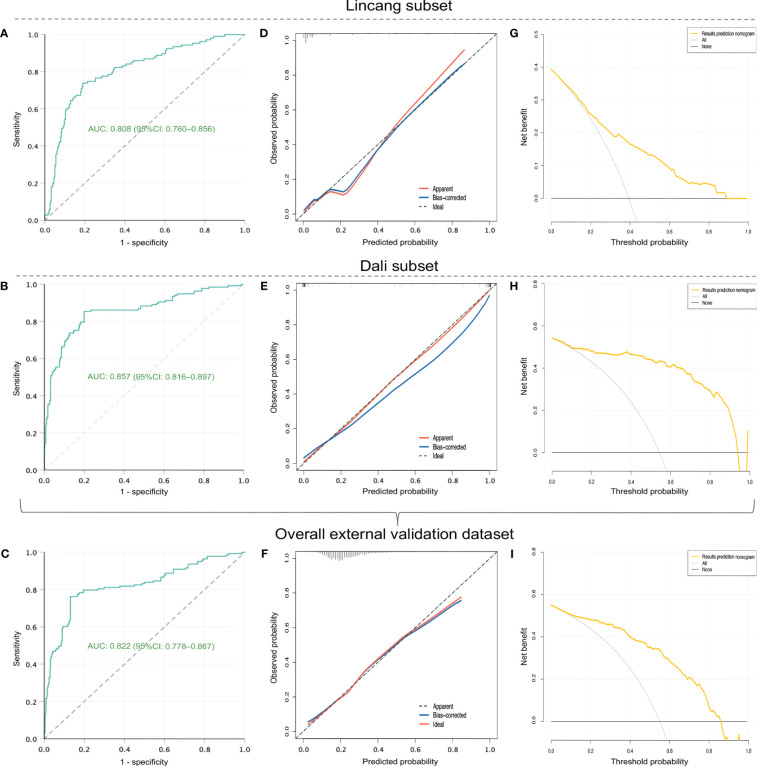
External validation of the nomogram in external validation dataset and its two subsets (Lincang and Dali subsets). **(A–C)** ROC curves. **(D–F)** calibration curves. **(G–I)** DCA. ROC, receiver operating characteristic; DCA, decision curve analysis.

A total of 249 RSV patients hospitalized at two other hospitals during January–March 2024 were included as the overall external validation dataset, comprising 142 cases in the Lincang subset and 107 cases in the Dali subset (**Table 2**). RSV-associated SALRTI was finally confirmed in 58 (23.3%) cases of these patients, whose characteristics were summarized in **Table 2**. The ROC curves of the nomogram showed an AUC of 0.808 (95%*CI*: 0.760–0.856, *P* < 0.001) in Lincang subset, an AUC of 0.857 (95%*CI*: 0.816–0.897, *P* < 0.001) in Dali subset, and an AUC of 0.822 (95%*CI*: 0.778–0.867, *P* < 0.001) in the overall validation dataset (**Figures 5A–C**), demonstrating a good discriminative ability. Meanwhile, the calibration curves presented favorable consistency between the predicted and actual probabilities of RSV-associated SALRTI in the overall validation dataset and its two subsets, since the bias-corrected curves were close to the ideal 45°curves (all *P* > 0.05) (**Figures 5D–F**). Besides, as shown in the DCA performed in the validation dataset and its subsets, the net benefits obtained from application of our nomogram within a wide range of threshold probability were greater than those from “all” and “none” schemes (**Figures 5G–I**), indicating that the nomogram had good clinical utility in predicting SALRTI in children hospitalized for RSV infection during the post-COVID-19 era.

**Table 2:** Characteristics of patients in external validation dataset.

Characteristics	Total (Overall dataset) (n=249)	Lincang subset (n=142)	Dali subset (n=107)
Age, months, median (IQR)	13.9 (9.4, 21.8)	13.5 (8.6, 20.8)	14.8 (10.2, 23.4)
Preterm birth, n (%)	34 (13.7)	19 (13.4)	15 (14.0)
Underlying condition, n (%)	38 (15.3)	20 (14.1)	18 (16.8)
Seizures, n (%)	26 (10.4)	14 (9.9)	12 (11.2)
NLR, median (IQR)	3.8 (2.9, 5.7)	3.9 (3.0, 5.9)	3.6 (2.7, 5.5)
IL-6, pg/mL, median (IQR)	20.8 (12.5, 36.9)	21.0 (12.8, 38.5)	22.3 (11.9, 34.3)
LDH, U/L, median (IQR)	403.5 (361.2, 568.9)	407.2 (371.4, 572.8)	398.7 (348.8, 554.6)
D-dimer, mg/L, median (IQR)	1.1 (0.7, 1.4)	1.1 (0.8, 1.5)	1.0 (0.7, 1.3)
Co-infection, n (%)	102 (41.0)	57 (40.1)	45 (42.1)
SALRTI, n (%)	58 (23.3)	35 (24.6)	23 (21.5)

IL-6, interleukin 6; IQR, interquartile ranges; LDH, lactate dehydrogenase; NLR, neutrophil-to-lymphocyte ratio; SALRTI, severe acute lower respiratory tract infection.

## 4 Discussion

In the present study, we developed and validated a novel, simple, and effective nomogram for predicting SALRTI in children hospitalized for RSV infection during the post-COVID-19 era. The nomogram was established based on the several inflammatory indicators and clinical characteristics, including age, preterm birth, underlying condition, seizures, NLR, IL-6, LDH, D-dimer, co-infection. This prediction model can be adopted by clinicians for accessing such patients due to its favorable performance verified by internal and external validations.

Of all the predictors, three variables, including age, preterm birth, and underlying condition were widely reported to be strongly linked with the severity of RSV infection before the emergence of COVID-19 (21, 22). Specifically, it is well acknowledged that pediatric patients accompanied by a history of prematurity and/or underlying diseases generally have higher risk of severe RSV infection, regardless of other physiological and pathophysiological factors (23). Besides, there has been abundant evidence demonstrating the close relationship between the age and severity of infection. The younger the age at infection, the higher the risk of severe RSV infection due to the weaker immune defenses in younger children (22, 24). However, it should be noted that despite a similar association between age and progression of RSV infection during the post-COVID-19 era, significantly changed age distribution was observed between the RSV patients in our study and those reported in previous literature. Children with RSV infection in the pre-COVID-19 era typically have a median age about 6 months, which is younger than that in our study (15.1 months). Foley et al. (25) also reported a similar phenomenon that the median age of pediatric RSV patients during the post-COVID-19 period in Australia was 16.4 months, more than twice that observed in the pre-COVID-19 era (8.1 months). A study conducted in China has attempted to investigate this phenomenon (13). They found that the levels of RSV-specific antibody among children were significantly declined during the post-COVID-19 era compared to those in the pre-pandemic era, and the changes of antibody levels showed prominent variability across different age groups, with a more significant decrease identified in older children. This might partially explain the trend towards increasing age of children with RSV infection during the post-COVID-19 era compared to the pre-pandemic period. Clinicians need to be aware of this marked change.

Notably, our study also identified a relatively unique predictor for RSV-associated SALRTI, i.e., seizures, which were uncommon in traditional RSV infection during the pre-COVID-19 period with an incidence of approximately 3.0–5.0% (26, 27). Whereas the incidence of seizures reached 11.9% across the overall development dataset and 17.2% in the severe group in our study, suggesting a sharp increase of RSV-associated seizures in post-COVID-19 era. Intriguingly, despite the low incidence of seizures in RSV infection during the pre-pandemic era, numerous studies have affirmed that for patients infected with SARS-CoV-2 similarly dominated by respiratory symptoms, seizures occurred more frequently in those with severe respiratory infections compared to those without (28–30). As recently reported by Proal et al. (31), virus might not be fully cleared in some individuals after recovering from acute SARS-CoV-2 infection. Instead, the replicating virus, viral RNA and/or viral protein can persist in tissue as a ‘reservoir’, which can cause neurological sequelae of COVID-19 (32, 33). Hence, the phenomenon of increased RSV-associated seizures during the post-COVID-19 era might be partially attributed to direct and/or indirect effect of the COVID-19 pandemic given the ultra‐high infection rate of SARS-CoV-2 during the pandemic and its clinical and biological characteristics. Seizures undoubtedly deserve more attention as they occurred more frequently in clinical course of RSV infection (especially for the severe cases) during the post-COVID-19 era than that during the pre-pandemic period.

In addition to the above clinical indicators, our study found that the increase of several inflammatory indicators (NLR, IL-6, LDH, D-dimer) were the independent risk factors for RSV-associated SALRTI. NLR is a novel hematological parameter for systemic inflammation and host immune response. In contrast to the counts of leucocyte or its subpopulations that showed relatively low sensitivity and specificity in the evaluation of inflammation and disease severity, NLR obtained by simple calculation (NLR = Neutrophil-to-lymphocyte ratio) has been increasingly suggested as one of the most effective and reliable biomarkers of immune-inflammatory response intensity and is gradually applied for the severity assessment of infection (34). Meanwhile, NLR was considered to be a relatively stable parameter that does not change dramatically with age and gender (35). Similarly, as a master regulator of inflammation, IL-6 is a typical pro-inflammatory factor that involves cytokine storm and inflammation responses central to the progression of infection diseases (36). During the process of RSV infection, innate immune cells play a fundamental role in response against RSV infection in the lower respiratory tracts. Pattern recognition receptors (PRR) on these cells can sense a series of target proteins of RSV, thus activating the nuclear factor kappa-light-chain-enhancer of activated B cells (NF-κB), interferon regulatory factors (IRFs), and mitogen-activated protein kinase (MAPK) pathway. As a consequence, the production and release of chemokines and inflammatory cytokines centered around IL-6 are ultimately triggered (2, 37). Noteworthily, multiple studies have shown that the combined application of IL-6 and NLR were more accurate than the single indicator in predicting inflammation intensity (38, 39). In the present study, NLR and IL-6 also demonstrated good predictive values for RSV-associated SALRTI.

In addition, LDH, a cytoplasmatic enzyme widely existing in tissues, is well recognized as a biomarker of cell damage, which can effectively monitor inflammation and progression of some diseases, including pulmonary/respiratory conditions (40). Subsequent to cell membrane damage, LDH would be leaked into bloodstream, resulting in an elevation of serum LDH level. Numerous previous studies have confirmed the important predictive value of serum LDH level in the severe progression of infectious diseases, such as COVID-19 and RSV infection (41, 42). As for D-dimer, it is the final product of the plasmin-mediated degradation of cross-linked fibrin and pronounced increase of D-dimer could be observed in individuals with serious viral infections (43, 44). The pathological increase of D-dimer may reflect inflammatory responses and activation of the coagulation cascade in the infectious state, implying the great potential of D-dimer for assessing the infection severity. Although adequate coagulation augmentation may function as a host’s defense mechanism against infection, excessive procoagulant activity is likely to cause fibrin deposition, exacerbating inflammation and tissue injury (45). In the prediction nomogram constructed in our study, LDH and D-dimer were also identified as powerful predictors for RSV-associated SALRTI, highlighting the essential roles of inflammation and potential tissue damage in the development and progression of this disorder.

Our study found that co-infection revealed significant predictive value for RSV-associated SALRTI during the post-COVID-19 era. In fact, RSV patients co-infected with other pathogens are also more likely to progress to severe conditions than those without even during the pre-COVID-19 period (46). This issue was more prominent in the post-pandemic era due to the influence of COVID-19-related mitigation measures that may induce immunity debt (6). During the post-pandemic era, not only has RSV experienced a drastic resurgence, but other pathogens (including influenza virus, human metapneumovirus, and adenovirus, etc.) have also undergone off-season outbreaks, accompanied by heightened severity (47). A large number of studies have reported increased incidence of mixed infection in patients with acute respiratory infection during the post-COVID-19 era, particularly in severe cases, as compared to the pre-pandemic period (16, 48). In line with these observations, the severe RSV group in our study showed more frequent co-infection than the non-severe group, and co-infection demonstrated strong predictive power for RSV-associated SALRTI. Therefore, the clinical management of pediatric RSV cases in this post-pandemic period necessitates particular vigilance in co-infection that showed an increased potential for exacerbating infection and leading to severe progression.

In the present study, nine variables, including age, preterm birth, underlying condition, seizures, NLR, IL-6, LDH, D-dimer, and co-infection were selected as the predictors for RSV-associated SALRTI in post-COVID-19 era, and were further integrated into a prediction nomogram. Intuitiveness is one important advantage of nomogram since it can transform each predictor of the model to a visual score, thus evaluating the risk of outcome by calculating a total point. Although previous studies have established some prediction model for severe RSV infection in children, it is necessary to construct new prediction tools to provide more help for clinical work due to the following two main reasons: on the one hand, the characteristics of RSV infecting have changed during the post-COVID-19 era compared to the pre-pandemic period (48, 49); on the other hand, previous prediction models often explored the predictive value of a single or two to three combined laboratory indicators for severe RSV infection, so the usefulness of these models in clinical practice might be limited (50–52). To cope with these, via analyzing the multicenter RSV data from post-COVID-19 era, our study constructed a more comprehensive nomogram covering common clinical and laboratory characteristics to provide more precise risk prediction of RSV-associated SALRTI. Meanwhile, despite an increased number of indicators required for our nomogram, this model still remained a good practicality as these nine variables in the nomogram are routinely measured and collected at admission for RSV inpatients. Because of that, the clinical application of this nomogram will not generate additional burden of variable collection and medical costs.

To fully assess the predictive accuracy and effectiveness of our nomogram, internal validation based on the development dataset and further external validation based on two independent pediatric RSV cohorts from different locations were performed. The final results of assessment showed a satisfactory prediction performance of this model. Furthermore, as suggested by DCA, the nomogram we developed was clinically applicable. Hence, based on this prediction model, early recognition of SALRTI in children hospitalized for RSV infection during the post-COVID-19 era may be achievable, assisting clinicians in medical decision-making. For RSV inpatients predicted as high risk of SALRTI, clinicians may choose earlier intervention or more aggressive therapies, or even transfer them to PICU for stricter monitoring and medical care, while those with non‐high risk might remain in the general respiratory wards for conventionally supportive treatments and monitoring. Early recognition and prevention of RSV-associated SALRTI is crucial for child health, especially given the increased severity and number of RSV cases during the post-COVID-19 era than the pre-pandemic period.

There were several limitations in our study. First, owing to the retrospective design, potential selection bias and confounders could not be fully excluded. In addition, although this was a multicenter study, the sample size was relatively small, and all subjects were enrolled from Yunnan province of China. Larger-scale prospective studies are warranted in the future to achieve higher accuracy and generalization of the prediction model.

## 5 Conclusion

Based on multicenter data, we established and validated a clinical and inflammatory indicators-combined nomogram for predicting the SALRTI in children hospitalized for RSV infection during the post-COVID-19 era. Nine strong predictors including age, preterm birth, underlying condition, seizures, NLR, IL-6, LDH, D-dimer, and co-infection were integrated into this model, which showed a good predictive ability, accuracy, and clinical usefulness.

## Data availability statement

The original contributions presented in the study are included in the article/supplementary material. Further inquiries can be directed to the corresponding author.

## Ethics statement

The studies involving humans were approved by Ethics Committees of Kunming Children’s Hospital. The studies were conducted in accordance with the local legislation and institutional requirements. The ethics committee/institutional review board waived the requirement of written informed consent for participation from the participants or the participants’ legal guardians/next of kin because Written informed consent was waived due to the retrospective design of the study.

## Author contributions

HL: Conceptualization, Formal analysis, Funding acquisition, Investigation, Methodology, Writing – original draft, Software. XZ: Formal analysis, Investigation, Software, Validation, Writing – original draft, Methodology. CL: Data curation, Formal analysis, Investigation, Methodology, Supervision, Writing – original draft, Project administration. WL: Data curation, Formal analysis, Investigation, Resources, Writing – original draft. W-HL: Data curation, Investigation, Software, Visualization, Writing – original draft. YW: Formal analysis, Investigation, Methodology, Resources, Validation, Writing – original draft. HL: Data curation, Formal analysis, Investigation, Resources, Writing – original draft. MX: Data curation, Formal analysis, Investigation, Methodology, Writing – original draft. RL: Data curation, Formal analysis, Investigation, Resources, Writing – original draft. TY: Data curation, Methodology, Software, Writing – original draft. HF: Conceptualization, Funding acquisition, Resources, Supervision, Writing – review & editing, Methodology, Project administration.
